# Modulations of neural activity in auditory streaming caused by spectral and temporal alternation in subsequent stimuli: a magnetoencephalographic study

**DOI:** 10.1186/1471-2202-13-72

**Published:** 2012-06-20

**Authors:** Ivan Chakalov, Rossitza Draganova, Andreas Wollbrink, Hubert Preissl, Christo Pantev

**Affiliations:** 1 Institute for Biomagnetism and Biosignalanalysis, University of Münster, Malmedyweg 15, 48149, Münster, Germany; 2 MEG-Center, Eberhard-Karls-University Tübingen, Otfried-Müller-Straße 47, 72076, Tübingen, Germany

## Abstract

**Background:**

The aim of the present study was to identify a specific neuronal correlate underlying the pre-attentive auditory stream segregation of subsequent sound patterns alternating in spectral or temporal cues. Fifteen participants with normal hearing were presented with series’ of two consecutive ABA auditory tone-triplet sequences, the initial triplets being the Adaptation sequence and the subsequent triplets being the Test sequence. In the first experiment, the frequency separation (delta-f) between A and B tones in the sequences was varied by 2, 4 and 10 semitones. In the second experiment, a constant delta-f of 6 semitones was maintained but the Inter-Stimulus Intervals (ISIs) between A and B tones were varied. Auditory evoked magnetic fields (AEFs) were recorded using magnetoencephalography (MEG). Participants watched a muted video of their choice and ignored the auditory stimuli. In a subsequent behavioral study both MEG experiments were replicated to provide information about the participants’ perceptual state.

**Results:**

MEG measurements showed a significant increase in the amplitude of the B-tone related P1 component of the AEFs as delta-f increased. This effect was seen predominantly in the left hemisphere. A significant increase in the amplitude of the N1 component was only obtained for a Test sequence delta-f of 10 semitones with a prior Adaptation sequence of 2 semitones. This effect was more pronounced in the right hemisphere. The additional behavioral data indicated an increased probability of two-stream perception for delta-f = 4 and delta-f = 10 semitones with a preceding Adaptation sequence of 2 semitones. However, neither the neural activity nor the perception of the successive streaming sequences were modulated when the ISIs were alternated.

**Conclusions:**

Our MEG experiment demonstrated differences in the behavior of P1 and N1 components during the automatic segregation of sounds when induced by an initial Adaptation sequence. The P1 component appeared enhanced in all Test-conditions and thus demonstrates the preceding context effect, whereas N1 was specifically modulated only by large delta-f Test sequences induced by a preceding small delta-f Adaptation sequence. These results suggest that P1 and N1 components represent at least partially-different systems that underlie the neural representation of auditory streaming.

## Background

The brain’s ability to constantly organize auditory objects or “auditory streams” from the competing sounds in the environment is a key element in human auditory perception. This phenomenon has been labeled *“stream segregation”* or “*streaming”* by Bergman and Campbell [1,2]. The most commonly-used stimulus configuration for investigating ‘streaming’, as described by Van Noorden [3], consists of a low-frequency tone A and high-frequency tone B presented in a sequence of repeated ABA triplets. When the frequency separation (Δf) between the tones is small and the inter stimulus interval (ISI) is long, the sequence is typically heard as one sound stream, like a gallop. In the case of a large Δf and brief ISI the galloping rhythm is no longer heard and, instead, the perception switches to two distinct sound streams of A and B tones [3,4]. At intermediate Δf and ISI values, the perception could bias in favor of one stream or two streams, depending on participants’ attention [3,5] and the duration of listening [6].

According to the “peripheral channeling hypothesis”, streaming occurs when different sounds excite adjacent peripheral channels with minimal overlapping in the cochlea and the auditory nerve [7]. However, the existence of additional acoustic cues that influence perceptual organization [6,8-11] as a function of time [2,6], suggest the contribution of centrally computed features in stream segregation [12-28]. An interaction between sub-cortical and cortical auditory structures in switching perception from non-streaming to streaming has been demonstrated recently [29].

Most electrophysiological studies interpret the neural correlates of streaming in terms of tonotopic organization of the auditory system [5,7,12,14,15,17-19,30-32], and Elhilali and colleagues demonstrated the critical role played by temporal coherence in the formation of auditory streams [33]. They showed that frequency-distant tones are no longer heard as distinct sound streams if presented synchronously rather than successively, although the enhanced neuronal evoked activity ascribed both sequences (synchronous and asynchronous) to streaming [33]. Thus, tonotopic organization itself is not enough to explain this type of perception; streaming requires both frequency separation and temporal incoherence [33]. In real acoustic environments, such as music and speech, however, sounds appear successively, and the perception and corresponding evoked activity of streaming are highly influenced by recent auditory experience [31,34,35]. Furthermore, the encoding of subsequent sounds can be influenced by attention [36,37] and distinct neuronal mechanisms are responsible for the automatic segregation of sounds and attention-dependent build-up processes [8,22,28,38].

The present study aimed to investigate the neural bases of auditory streaming at a pre-attentive level by making alterations to the spectral or temporal features of succeeding sound sequences. Our experiments were motivated by the hypotheses that selective adaptation underlies stream segregation [15,16,23] and that modulations in auditory evoked activity can indicate this process [16,21,22,35,39]. Since streaming is based on adaptation to specific tone frequencies [14-16] and is strongly determined by the ISI [40], it seems reasonable to expect the encoding of subsequent streaming patterns to rely on similar physiological mechanisms. Furthermore, multiple levels of representation in auditory stream segregation have been proposed by psychophysical comparisons of the effects of different types of initial adaptation stimuli [23,34]. Neuronal activity in streaming is also known to be modulated by prior perception [28,35]. In an event-related potential study by Sussman & Steinschneider, for instance, they showed the absence of mismatch negativity to a ‘deviant tone’ (a tone which deviated in frequency) from their repeating ABBB Test sequence induced by a priming sequence [28]. Neurological responses to the deviant tones are thought to arise during streaming and thus the authors concluded that no streaming occurred when the Test sequence was preceded by a smaller ∆f Adaptation sequence. No behavioral responses were collected, however, so comparison of behavioural reponses with evoked activity was not possible[28]. Another EEG research by Snyder and colleagues found that the adaptation to a prior stimulus pattern could underline the effects of functionally distinct neural processes in stream segregation [35]. They also demonstrated that a large ∆f in the initial Adaptation sequence results in reduced streaming on the subsequent Test sequence and vice versa [35].

The current study tested the hypotheses that frequency-selective adaptation underlies stream segregation and that prior adaptation stimulation could recover specific neural correlates and streaming effects. We measured the auditory evoked fields (AEF) in response to an ABA triplet paradigm consisting of successive prime Adaptation and subsequent Test sequences. The contextual effect between consecutive tone patterns is known to decrease as the period of silence between them increases [2,31,35,40,41], therefore the neurophysiological demonstration of a clear adaptation effect requires continual stimulation. For this reason we chose to present our experimental conditions as an uninterrupted trial (without silence between the sequences). In order to distinguish between adaptation-based effects and those modulated by attention, the participants were instructed to concentrate on a soundless movie of their choice and ignore the ongoing auditory stimulation. We set up two consecutive MEG experiments to manipulate either the frequency separation (Δf) or the inter stimulus interval (ISI) of the succeeding adaptation and test sequences. In addition, behavioral measurements were performed, whilst replicating all sessions of the MEG study, in order to evaluate the participants’ perceptual state.

In the *first* experiment, Δf between the A and B tones of the initial Adaptation and subsequent Test sequences was altered (small, intermediate or large Δf) by varying the frequency of the B tones. The frequency of the A tones did not vary throughout the entire experiment. We anticipated that the presentation of a small ∆f during the Adaptation sequence would induce increased neuronal activity during a subsequent intermediate or large ∆f Test sequence because of the concomitant reduction in neuronal adaptation as Δf increases at the beginning of the Test sequence. We also expected to see the complimentary result, that large and intermediate ∆f values during the Adaptation sequence would result in decreased neuronal activity during the small ∆f Test sequence. The ISI between A and B tones did not vary during this first trial.

In the *second* experiment, a constant ∆f of 6 semitones was used and the ISI was varied. A short ISI in the Adaptation sequence was followed by a long ISI in the Test sequence and vice versa. In this experiment we specifically investigated whether the undergoing adaptation effect is dependent upon the specific frequency of the A or B tone, or whether particular neurons might be tuned to a specific ∆f-shift, regardless of the actual frequency range [2,3]. The process of adaptation in response to simple frequencies in the auditory system is known to be dependent on the concrete repetition rate of the stimuli [15,16]. Thus, if the expectations proposed above (for Experiment 1) rely on adaptation to the repeated presentation of the A and B tones, changing the rate of presentation between the two sequences should disrupt the adaptation course and not induce modulation of the neuronal activity during the Test-sequence in that experiment.

## Methods

### Test participants

Fifteen right-handed participants (5 males) aged between 22 and 30 years participated in this study. None of them had a history of otological or neurological disorders. Normal audiological status, defined by air conduction hearing thresholds of less than 10 dB HL in the frequency range between 250 and 4000 Hz was verified by pure tone audiometry. Participants gave written informed consent to take part in the study in accordance with procedures approved by the Ethics Commission of the University of Münster and the Declaration of Helsinki.

### MEG experiments

**Figure 1:**
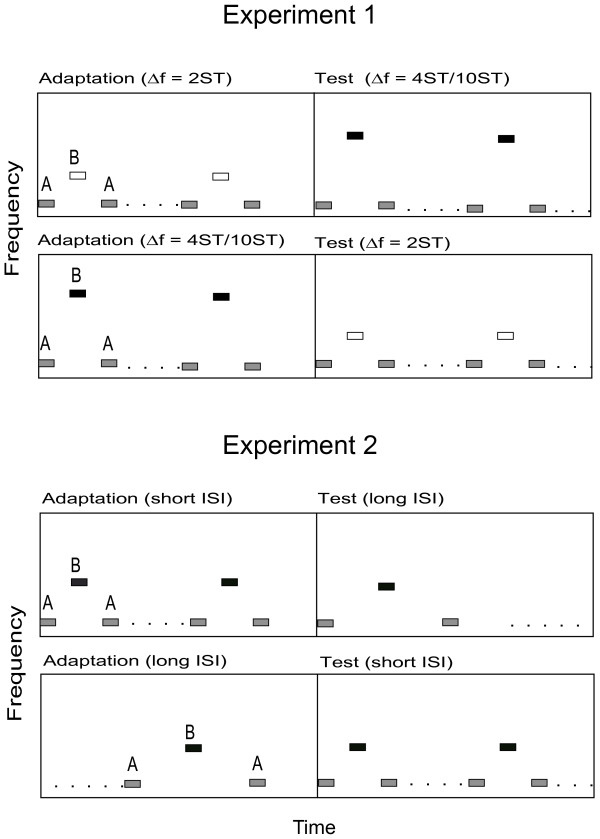
**Experimental design.** This figure illustrates the two different ABA sequences (Adaptation and Test) presented in Experiments 1 and 2, plotted as relative Frequency against relative Time. These sequences were presented with randomly-organized changes in position in uninterrupted trials. (**A**) **Experiment-1;** The Inter Stimulus Interval (ISI) between A and B tones in both sequences was fixed at 125 ms. Three different degrees of Δf (2, 4 and 10 semitones) were presented as either Adaptation or Test sequence. (**B**) **Experiment-2;** Δf between successive A and B tones was fixed at 6 semitones but the ISI varied between short (100 ms) and long (225 ms).

Two different MEG experiments were carried out using ABA tone-pip sequences (sinusoidal tone-pips of 25 ms duration, including 10 ms rise and decay times). The loudness of the stimuli was set to 60 dB above the individual hearing thresholds. Trials were organized as a combination of the Adaptation sequence and subsequent Test sequence in an uninterrupted trial. In two subsequent experiments we investigated the effects of the preceding Adaptation sequence on the following Test sequence by varying (a) frequency separation (Experiment 1) and (b) inter stimulus interval (Experiment 2) (c.f. Figure 1). The duration of each trial was 12 s: 6 s for the adaptation and 6 s for the following test sequence. In each experiment, 80 trials were presented: 40 Adaptation sequences and 40 Test sequences. The ordering of the Adaptation and Test sequences was randomly organized. The inter trial interval (ITI) was 3 s, thus the total recording time of one experimental session was 20 minutes.

**Experiment 1** consisted of two 20-minute sessions during which three different ∆f values for the ABA triplets were presented: small (2 semitones), intermediate (4 semitones) and large (10 semitones). In each case the frequency of tone A was 500 Hz; therefore tone B was either 561 Hz for the small ∆f, 630 Hz for the intermediate ∆f or 891 Hz for the large ∆f, respectively. The ISI linking the ABA tones as triplets was constantly set to 100 ms; thus the ISI between successive A tones was 225 ms and between successive B tones was 475 ms.

In the first session a small ∆f Adaptation sequence was followed by a intermediate ∆f Test sequence and vice versa (c.f. Figure 1A), with the exact ordering of these conditions randomized. The second session compared small ∆f Adaptation with large ∆f Test sequences, and vice versa, in the same way.

**Experiment 2** consisted of one 20-minute session in which the ISIs between A and B tones in consecutive sequences were changed, opposing long ISI vs. short ISI and vice versa (c.f. Figure 1B). ∆f remained at 6 semitones (A = 500 Hz and B = 707 Hz) throughout Experiment 2, a value that has been previously determined in many studies to facilitate the perception of two streams [16,21,22,34,35]. In the long ISI condition, the period between the ABA triplet tones was 225 ms; thus, the ISI of successive A tones and successive B tones remained at 475 ms and 975 ms, respectively. In the short ISI condition, the A and B tones were presented twice as fast, with an ISI of 100 ms between triplet tones. The ISIs between successive A and successive B tones were therefore 225 ms and 475 ms, respectively. In the same manner as in Experiment 1, a short ISI-Adaptation sequence was followed by a long ISI-Test sequence and vice versa (long ISI followed by a short ISI), with the exact ordering of these conditions randomized.

### Behavioral experiment

In the behavioral experiment we used the same experimental design as the MEG measurements (described above, also c.f. Figure 1) to investigate the participants’ perceptual state. Three participants from the prior MEG-measurements could not participate in the behavioral tests. Three sessions were carried out, corresponding to both MEG-experiments. In the first session a small ∆f Adaptation sequence was followed by a intermediate ∆f Test sequence and vice versa, in randomized order (as in session one of Experiment 1). The second session compared small ∆f Adaptation with large ∆f Test sequences, and vice versa (as in session two of Experiment 1). In the third behavioral session a short ISI Adaptation sequence was followed by a long ISI Test sequence and vice versa (as in Experiment 2). All three 15 minute sessions took place on the same day two weeks after the MEG-measurements. Within each session 24 trials were presented, corresponding to 12 repetitions of each of the conditions (small versus intermediate ∆f; small versus large ∆f; short versus long duration ISI). In order to avoid the effect of “echoic memory” involving successive trials of active listening [34,42-44], the trial duration was shortened to 8 s (4 s of Adaptation and 4 s of Test) and the inter trial interval (ITI) was extended to 5 s. The participants were instructed to indicate the point at which their perception switched from one to two streams by pressing a mouse button during the presentation of each sequence. Prior to the experiment, the participants practiced 4 sequences of each condition in order to restrict intentional biasing of the perception in favor of one of the streams (A or B tones) [3,5]. The responses to each condition in each position as Adaptation or Test sequence were collected separately across all three sessions.

### MEG data acquisition

The MEG recording was performed using a 275-channel whole-head system (Omega2005, VSM-Medtech, Port Coquitlam, BC, Canada), sampled at 600 Hz. The MEG measurements preceded the behavioral recordings in order to avoid possible effects of attention, intention and prior experience. The participants had not received prior information about the paradigm, the stream segregation phenomena or the purpose of the study. They were comfortably seated in an upright position, instructed to ignore the sounds and watch a soundless video of their choice, presented on a monitor in front of them [16,22]. Eighty trials per session were recorded, yielding a sufficient number of MEG data.

### Data analysis of MEG data

The MEG analysis was performed using the CTF software package. After applying a high-pass filter with cutoff-frequency of 1 Hz the MEG data were separated into epochs of 450 ms (from −50 to +400 ms relative to the stimulus onset of the ABA triplets). Epochs with magnetic field amplitudes (peak-to-peak) larger than 3 pT were excluded from further analysis as artifacts. After averaging, a 20 Hz low-pass filter was applied. Importantly, the responses to the first and last triplets in each condition were excluded from the averaging procedure in order to avoid a stronger influence from the onset and offset of each sequence on the grand average data.

Assuming the model of an equivalent current dipole (ECD) in a spherical volume conductor, a spatio-temporal dipole fit was performed. T1-weighted magnetic resonance imaging (MRI) of the head was obtained from all listeners on a 3 T Scanner (Gyroscan Intera T30, Philips, Amsterdam, Netherlands). The parameters (center location and radius) of the spherical head model were derived from the individual MRI. The interval used for the ECD fit (~30 ms) was placed around the local maximum of the P1 component of the AEF. The P1 dipolar sources evoked by tone B were less variable across conditions compared to the N1 sources and, thus, provided a better signal-to-noise ratio. For each participant, two ECDs (one in each hemisphere) were determined, defined by their dipole moment, orientation and spatial coordinates (a goodness of fit larger than 85% was imposed). Median values of x, y, and z coordinates of the dipole location and the angles of the dipole orientation were calculated for each condition. The individual averaged values for P1 (across all conditions) were used to fix the dipole position and orientation and then the source space projection method [45] was applied to calculate the components of the transient evoked response (P1, N1) for each condition.

### Statistical analysis of MEG data

The major goal of this study was to examine the influence of the initial stimulation on the Test sequence condition. For that purpose, the responses of the same condition presented in Adaptation and Test positions were compared (cross-checking comparison).

Repeated measurements 2x2x2 ANOVA were performed for the peak amplitudes and latencies of P1 and N1 components to the B tones of the ABA triplet. The statistical analysis always included three factors: "Hemisphere” (right (RH) and left (LH)), "∆f " (Experiment 1) or "ISI" (Experiment 2) consisting of 2 variables for each of the investigated ∆f values (∆f = 2 vs. ∆f = 4 or 10 semitones, or short ISI vs. long ISI) and “Part” (also containing two variables, showing that the stimulus was presented as an Adaptation or Test condition). For two participants, P1 (one participant) and/or N1 components (one participant) could not be derived from the average response time course, and were not included in the group analysis.

Four separate ANOVAs were performed for Experiment 1 and one further separate ANOVA for Experiment 2. For all statistical analyses Bonferroni correction was applied.

In the first analyses the 2 and 4 semitone conditions from the first session of Experiment 1 were examined. This enabled us to test the effect of the initial 4 semitones condition on the subsequent 2 semitones Test and the effect of the initial 2 semitones on the 4 semitones Test by crosschecking the effects of the identical condition presented in Adaptation and Test positions. Another separate ANOVA was applied for the second session, to investigate the effect of 2 semitone Adaptations on the 10 semitone Test and vice versa. In the third analyses, the initial effect of the 2 semitone conditions on the 4 and 10 semitone Tests was examined. For that purpose, the 4 and 10 semitone conditions from the first and second sessions, presented in both Adaptation (for control) and Test positions were entered into another ANOVA. In a fourth ANOVA only the 2 semitones Test conditions from session one (following an initial 4 semitones) and session two (initial 10 semitones) of Experiment 1 were entered. In that way the initial effects of 4 and 10 semitone Adaptation conditions on the neural activity of the 2 semitone Test were investigated. In this analysis “∆-f” was labeled “session” (2 semitone condition from session 1 and from session 2).

A separate ANOVA-analysis was also used for Experiment 2, in the same way as for the first experiment.

## Results

### Source waveform data

#### Experiment 1*. Frequency-based neuronal activity and lateralization effects*

**Figure 2:**
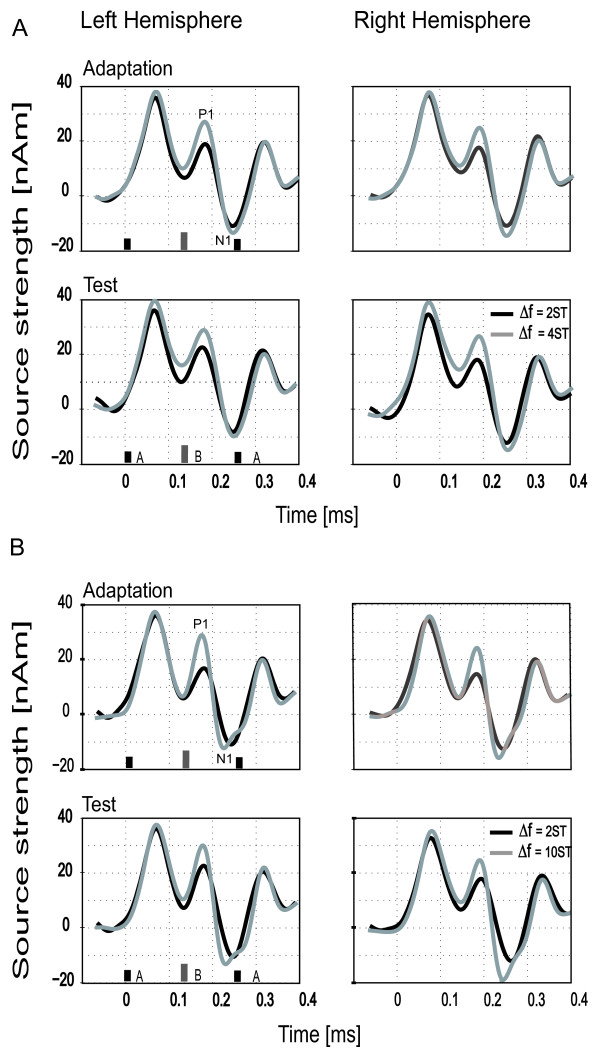
**Grand averaged source waveforms across all participants from Left and Right Hemispheres.** (**A**) The two different degrees of frequency separation between A and B tones (Δf = 2 semitones [black lines] and Δf = 4 semitones [grey lines]) which were presented in the first experimental session are shown. The responses recorded from their presentation as Adaptation (on top) or Test (below) sequences are shown. (**B**) The second experimental session presented stimuli of Δf = 2 (black lines) and Δf = 10 semitones (grey lines), presented as Adaptation (top) and Test (below) sequences.

The grand averaged responses from the first session of Experiment 1, are shown in Figure 2A (small ∆f [2 semitones] are indicated by black lines; intermediate ∆f [4 semitones] are in gray). The upper and the lower graphs display the responses to the Adaptation and Test sequences, respectively. The amplitude of the P1 component was generally found to have increased for ∆f = 4 semitones compared to ∆f = 2 semitones (significant main effect ∆f [*F*(1,14) = 23.468, *p < .001]),* whereas the amplitude of the N1 component was only slightly stronger in ∆f = 4 semitones compared to ∆f = 2 semitones. The P1 amplitudes were also slightly larger in the left compared to the right hemisphere. In contrast, the N1 amplitude was significantly enhanced in both conditions (4 and 2 semitones) in the right hemisphere compare to left hemisphere only on a Test position (interaction Hemisphere x Part for N1 amplitude [*F*(1,14) = 13.993, *p* < .05]).

Figure 2B shows the grand average responses from the second session of Experiment 1, with the P1 component significantly stronger in condition ∆f = 10 semitones compared to the ∆f = 2 semitones (significant main effect ∆f [*F*(1,13) = 12.598, *p* < *.001]).* This enhanced amplitude was more prominent when presented as a Test sequence (main effect “Part” for P1 amplitude [*F*(1,13) = 5.610, *p* < *.05] ).* The N1 amplitude was also significantly enhanced when the 10 semitones condition was presented as a Test sequence and it was more dominant in the RH (significant interaction ∆f x Part for N1 amplitude [*F*(1,13) = 8.384, *p < .05]* and main effect of Hemisphere [*F*(1,13) = 8.542, *p < .05]).* In addition, the N1 was significantly shortened in the RH for both 2 and 10 semitones (significant main effect Hemisphere for N1 latency, [F(1,13) = 6.093, p < .05)]).

The responses of P1 and N1 for 4 semitones and 10 semitones conditions from the first and second sessions of Experiment 1 (in adaptation and test position) were entered in another separate 2x2x2 model ANOVA, in order to compare the effects induced by the preceding small ∆f sequence (2 semitones). The statistical analysis showed that P1 was significantly increased in the LH for both ∆f = 4 and ∆f = 10 semitones conditions presented as Test sequence, compare to adaptation position (significant interaction for Hemisphere x Part [*F*(1,13) = 11.747, *p < .05]*)*.* In contrast, the N1 amplitude was increased in the RH for these same conditions (significant main effect Hemisphere [*F*(1,13) = 6.759, *p < .05]).* This lateralization effect was more prominent for ∆f = 10 semitones during the Test sequence (significant interaction Hemisphere x ∆f [*F*(1,13) = 8.851, *p < .05],* and ∆f x Part [*F*(1,13) = 8.044, *p < .05]*)*.* For the 4 and 10 semitones ∆f stimuli presented only on adaptation position (no initial stimulation), significant effects were obtained regarding the lateralization of the amplitude of N1 (main effect Hemisphere [*F*(1,14) = 7.318, *p < .05]* and interaction Hemisphere x ∆f [*F*(1,14) = 6.676, *p < .05]),* the N1 was significantly larger in the LH for *∆*f = 4 semitones, whereas for *∆*f = 10 semitones the amplitude was stronger in the RH.

A further separate ANOVA was applied to data from the first and second sessions in order to test the effects of an initial intermediate (∆f = 4) and large (∆f = 10) frequency separation on the small ∆f Test condition. Only the amplitude of P1 was significantly enhanced when the 2 semitone stimulus was presented in the Test position condition compared to Adaptation position (significant main effect Part [*F*(1,13) = 6,331, p < .05]).

**Figure 3:**
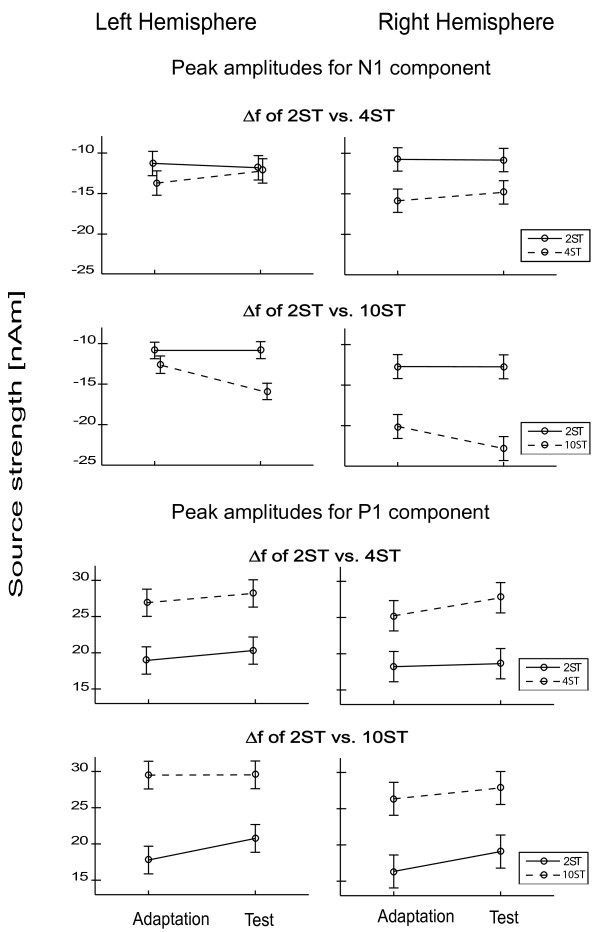
**Peak amplitudes of N1 and P1 from each hemisphere to all three degrees of frequency separation.** This figure shows responses tested across the two sessions which constituted Experiment 1 (Δf = 2 vs. 4 semtones; Δf = 2 vs. 10 semitones) presented as Adaptation and Test sequence. Error bars represent 95% confidence intervals. The upper plots show N1 responses and the lower plots show P1 responses.

Figure 3 summarizes the fluctuations in neuronal activity and the lateralization effects for all ∆f values (2, 4 and 10 semitones), presented as Test or Adaptation, separately for the P1 and N1 components. Error bars indicate the 95% confidence intervals for the within-subject effect (Part x Semitones), by Loftus & Masson [46].

#### Experiment 2*. Influence of the alternating ISI*

**Figure 4:**
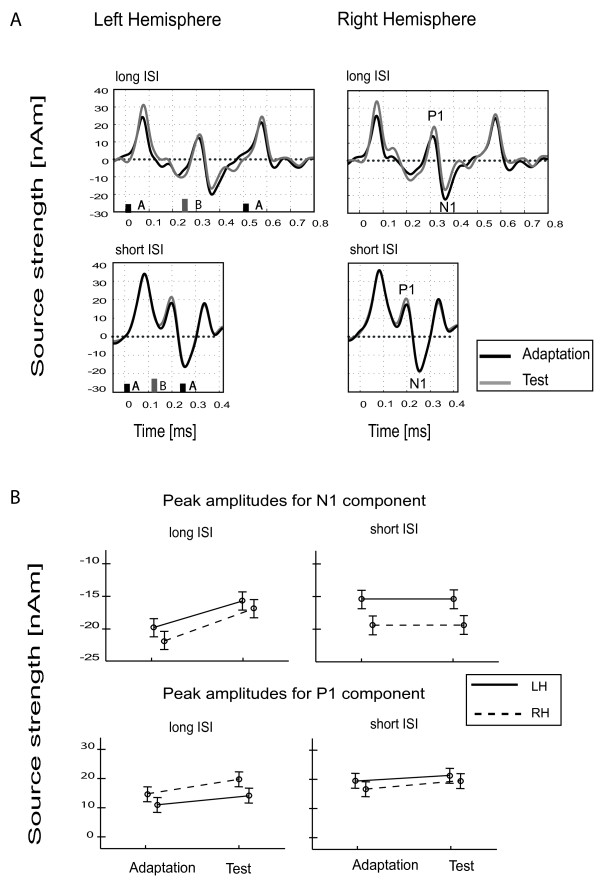
**The grand averaged source waveforms for the two different ISIs used in Experiment 3, plotted as Source Strength (nAm) against Time (ms). **(**A**) Long ISI is shown at the top and short ISI at the bottom, with the Adaptation marked by black lines and the Test in grey. The frequency difference was fixed at 6 semitones in both conditions. (**B**) The averaged peak amplitudes of N1 (top) and P1 (bottom) obtained in Test and as Adaptation sequence positions with the Left hemisphere and Right hemisphere shown as solid or dotted lines, respectively.

The short ISI condition was analyzed in the time-interval of −50 ms to 400 ms and the long ISI condition was analyzed in the time-interval of −50 ms to 800 ms. The results of these analyses are displayed in Figure 4A with Adaptation and Test conditions denoted by black and grey lines, respectively. Concerning the B-tone, significant main effects and interaction regarding the position in the trial and the lateralization were neither obtained for P1 amplitude (main effect Hemisphere [*F*(1,13) = 1.397, *p =0 .258],* ISI [*F*(1,13) = 2.036, *p =0 .177],* Part [*F*(1,13) = 3.201, *p =0 .097]* and Hemisphere x Part [*F*(1,13) = 2.431, *p =0 .143])* nor for N1 amplitude (main effect Hemisphere [*F*(1,13) = 2.024, *p =0 .178], ISI* [*F*(1,13) = 0.604, *p =0 .451],* Part [*F*(1,13) = 0.640, *p =0 .438]* and Hemisphere x Part” [*F*(1,13) = 3.755, *p =0 .075]*).

Figure 4B shows the neuronal activity and the lateralization effects for short ISI and long ISI, separately for P1 and N1 components, presented as Test or as Adaptation. Error bars represent 95% confidence intervals for the within subject effect Part x Hemisphere [46].

### Behavioral data

**Figure 5:**
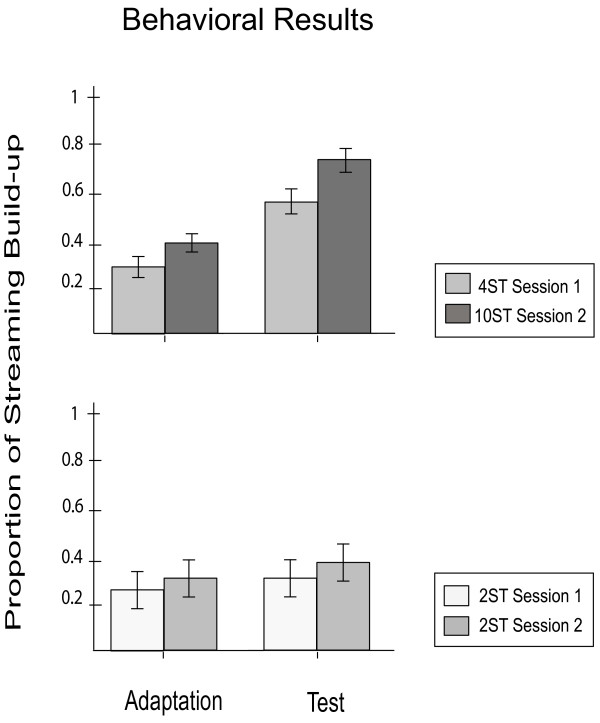
**The proportion of trials perceived as streaming in the Behavioral experiment, illustrating the effect of the initial Adaptation on the streaming perception of the subsequent Test.** The Upper section plots the averaged proportion of trials in the 4 and 10 semitones condition which were perceived as two streams when presented in the Test position (proceeded by the 2 semitone stimuli), allowing comparison with the perception of the same stimuli presented in the Adaptation position. The Lower section plots the proportion of trials heard as two streams in the 2 semitones scenario when presented as both Test (preceded by 4 and 10 semitones) and as Adaptation sequences. Error bars represent the standard error of the mean.

The results of the behavioral experiments are shown in Figure 5. The data obtained in response to small ∆f (2 semitones) from the first two sessions were analyzed separately from the intermediate and large ∆f conditions (4 and 10 semitones). The statistical analysis revealed significantly better performance of streaming when ∆f of 4 and 10 semitones stimuli were presented in the Test position compared to the same stimuli presented in the Adaptation position (significant main effect Part [*F*(1,11) = 14.286, *p < .05]).* In the third session no participants reported the perception of two separate streams in the long ISI condition, regardless of its presentation as Test or Adaptation sequence. In contrast, all participants perceived two streams in the short ISI condition but there was no significant difference related to the position as Test or Adaptation sequence [*t*(11) = −1,149, *p* = 0. 275; independent sample *t* test].

## Discussion

### Neurophysiologic correlates of stream segregation

#### Effect of alternating the frequency separation (Experiment 1)

To our knowledge, this study reports for the first time the differing behavior of P1 and N1 components of human AEFs when the frequency separation between A and B tones during initial Adaptation and subsequent Test sequences of a streaming task have alternated. Neural activity generally increased as the frequency separation between A and B tones increased, a finding which is in line with previous studies [16,21,22,35]. We found that in all conditions (∆f of 2, 4 and 10 semitones) the P1 enhancement was more prominent during the Test sequence but that the N1 amplitude was significantly increased only in the 10 semitone Test-condition.

Neuronal activity underlying streaming is known to be modified by prior adaptation [28,35], but the reported modulations of P1 and N1 components are not as specific, as seen here. Snyder and colleagues, for instance [35] performed a similar EEG study with a two-consecutive-conditions design but with 1.44 s of silent interval between conditions. The Δf of the Adaptation sequence in their study was 3, 6, or 12 semitones, followed by a Test sequence with a Δf of 6 semitones. Unlike our design, the participants were required to pay attention to the stimuli. They reported that a small Δf in the Adaptation sequence induces P1 enhancement in response to the first A tone of the ABA triplet in the Test sequence. In the same manner, in our study the P1 component was enhanced in all conditions on a Test position, therefore the P1 clearly shows the context effect. We also found a significant enhancement of the N1 component in the case of the 10 semitone Test condition following the initial 2 semitones Adaptation sequence. This further modulation of the N1 could have been induced by the specific paradigm we used (two Adaptation and Test-sequences with no gap in between), because the contextual effect between consecutive tone patterns has been shown to depend upon the period of silence between them [2,31,40,41] and to decrease as a function of time [35]. Furthermore, the enhancement of neural activity which we observed during the intermediate and large ∆f Test sequences following adaptation to a small ∆f reliably concurred with our predictions the implications of previous research [35]. However, the amplitude of the P1 component was also found to increase in the small ∆f-Test sequence following initial larger ∆f stimulation, despite our expectation that neuronal activity would reduce. The P1 component therefore appeared to be enhanced in all Test conditions, regardless of the size of the initial ∆f Adaptation. This finding is contrary to previously reported psychophysical results [23] that demonstrated a reduced probability of two streams being perceived after adaptation to a larger ∆f pattern. On the other hand, Snyder and colleagues [23,34] proposed that multiple levels of representation could underlie the perceptual organization of sequential auditory patterns, and the facilitative perceptual effect and the stimulus driven-effect are proposed to be at least partially independent [34]. It has also been suggested that distinct cortical areas may be active in the encoding of the adaptation and test streaming patterns [35]. Given this suggestion, it is likely that the P1 and N1 response components represent different modulation effects in the encoding of subsequent streaming patterns: P1 demonstrates the adaptation effect (context) and N1 demonstrates the streaming effect. Further support for this assumption is in the dissimilar task sensitivity of both components [47], and this is in turn supported by evidence that P1 and N1 are generated by different auditory cortical regions [48-51]. Moreover, distinct neuronal mechanisms are responsible for automatic auditory stream segregation and attention-dependent buildup processes [8,22,28,38], and attention operates in a more pronounced manner on N1 and later components (P2 and N2) [36,51-53]. For this reason, additional modulation in the auditory cortical network could be expected in an active listening task.

### Lateralization effects, based on frequency alternations

Our results from the first experiment showed lateralization-differences of the B tone-related P1 and N1 components between different frequency separation conditions (2, 4 and 10 semitones). The amplitude of the P1 component was significantly increased for all conditions (Δf of 2, 4 and 10 semitones) in the LH during the Test sequence. In contrast, the N1 amplitude was significantly enhanced in the RH for higher frequency separations only (Δf of 4 and 10 semitones). The N1 latency was also shortened in the case of the 10 semitones stimulus in the Test, which is in line with the findings of Roberts & Poeppel who demonstrated that the N1 peak latency varies as a function of the stimulus frequency [54]. It has been also proposed that amplitude and latency lateralization of the N1 component has a common origin [55]; a proposal which can also be observed in our data (RH amplitude and latency lateralization of N1). Regarding streaming, Snyder and colleagues demonstrated that the modulation of N1 occurred at the right but not at the left temporal electrode, suggesting an RH dominance for frequency separation-based segregation [22], in line with our findings.

In summary, the differences in the modulation of the AEFs that were demonstrated in the first experiment suggest that P1 and N1 represent at least partially-different mechanisms both of which are active during the automatic segregation of subsequent streaming patterns. The two components also showed different lateralization behavior: P1 was commonly enhanced in the LH in all conditions, whereas N1 was increased significantly only in the RH during the 10 semitone Test sequence.

#### Effect of alternating the ISI (Experiment 2)

The goal of the second experiment was to test the effect of alternating the ISI between A and B tones in consecutive Adaptation and Test conditions in an uninterrupted trial. The frequencies of A and B tones were kept constant during the whole trial; therefore the tonotopic representation was identical. The morphology of the AEF responses to the short ISI condition in both the Test and Adaptation positions was almost identical and not significantly different in comparison to long ISI responses. Thus, the presentation of an initial long ISI condition did not influence the segregation process during the following short ISI sequence and vice versa. Since adaptation in the auditory cortex is dependent not only on the specific frequency but also on the concrete repetition rate of the stimuli [13,56,57], it could be suggested that the different ISIs which were used in the consecutive Adaptation and Test sequences caused disruption of the frequency-specific adaptation to A and B tones. Both sequences were therefore processed as distinct temporal events, without the contextual influence of the neural activity of one upon the other, regardless of having the same tonotopic representation. Based on that finding, it could be suggested that the modulation effects reported in the first experiment reflect the repeated presentation of specific tone frequencies but not of neurons tuned to a certain ∆f-shift [3]. This idea has also been tested in a recent psychophysical study by using Adaptation sequence A tones of greatly different frequency to those of the Test sequence, and B tones of either 3 or 12 semitones above the A tone frequency [23]. The presentation of a small or large Δ*f* initial sequence did not affect the perception of streaming during the test, supporting the idea that the context effect reflects adaptation to the repeated presentation of specific tone frequencies [23]. It should be mentioned, however, that the ISI values used in the present experiment were extreme (one very long ISI and one half the length) and that these conclusions regarding the concrete ISIs and modulation of the neural activity are therefore not necessarily applicable to other ISI conditions.

### Behavioral study

The behavioral study was conducted in order to evaluate the participants’ perception using the same paradigm as in the MEG measurements. All aspects of the previous MEG experiments were replicated as well as the behavioral measurements. The results revealed that the likelihood of reporting two streams increased as Δf increased, a finding which is in line with previous studies [16,21,22,35,39]. The main purpose was to evaluate the context effects of initial adaptation on the streaming perception during the Test sequence, in order to compare them with the neurophysiological findings. The results demonstrated a greater probability perceptual streaming in response to large and intermediate Δf in the Test sequence position following a small Δf Adaptation sequence compared with a large or intermediate Δf on Adaptation position. No significant effects or interactions were found regarding the perception of small Δf Test sequence when preceded by large (10 semitones) and intermediate (4 semitones) Δf Adaptation presentations despite the fact that the P1 component appeared to be enhanced in the MEG outcome of our study. The lack of perceptual modulation during the 2 semitone Test sequence could have been caused by using a small Δf because the perceptual context effect was reported to be strongest at intermediate Δf values [23,34,35]. Furthermore, it has been proposed that streaming build-up occurs at levels of neuronal representation with sharp frequency tuning, unlike the effect of prior context which is caused by the adaptation of neurons with wide frequency tuning [23]. Thus, modulations in neural activity were reported in our neurophysiological experiments in all Test conditions (enhanced P1 component), unlike the perceptual effect which was less responsive to the initial large Δf Adaptation conditions.

The behavioral data obtained when altering the ISI, showed no significant differences in the short ISI Test condition when preceded by the long ISI Adaptation sequence and vice-versa. We therefore conclude that adaptation sequences of different ISIs do not affect the subsequent perception of streaming. This psychophysical finding compliments the results of the corresponding MEG experiment which showed no AEF modulation following variation of ISI in the Adaptation sequence. As proposed above, the neurons undergoing the adaptation effect are sensitive not only to a specific frequency range, but also to the concrete repetition rate of the presenting stimuli [13,56,57]. Our findings lend further support to the idea that the initial adaptation that facilitates the streaming process is rather a peripheral mechanism that depends on simple tone frequencies but not higher order auditory features [23].

In addition, it should be mentioned that the perceptual state cannot be directly ascribed to the electrophysiological data seen in prior pre-attentive MEG experiments, since the buildup of streaming requires several seconds [2,31]. Moreover, different levels of representation underlying the contextual influence in auditory stream segregation have been suggested by Snyder and colleagues [23] as pointed out above, and the effect of the initial context that facilitates the streaming perception appeared to be different from the neural effect caused by the prior small or large Δf-based adaptation [28,35].

## Conclusions

Our MEG experiment demonstrates dissimilar behavior in P1 and N1 response components during the automatic segregation of sound, induced by an initial Adaptation sequence. The P1 component appeared to be enhanced in all Test conditions and thus demonstrates the preceding context effect, whereas N1 was specifically modulated only in the large frequency separation Test condition induced by a preceding small Δf Adaptation sequence. This finding combined with the difference we observed in the lateralization of P1 and N1 could suggest that both components represent at least partially-different systems underlying the perceptual organization of streaming patterns. The psychophysical results of the parallel behavioral study show that prior adaptation to a smaller degree of frequency separation facilitates the streaming perception. However, neither the neural activity nor the perception of successive streaming sequences were modulated when the inter stimulus intervals were varied, thus lending support to the idea that the initial modulation effect reflects adaptation to the repeated presentation of specific tone-frequencies.

## Competing interests

The authors declare that they have no financial or any other competing interests.

## Authors’ contributions

IC, RD and CP conceived of the study designed the experimental setup and the auditory stimuli. IC and RD acquired the data. IC performed the data & statistical analyses. All authors participated the data evaluation and interpretation and in writing the manuscript, and have approved the final version of the manuscript.

## References

[B1] Bregman AS Campbell J Primary auditory stream segregation and perception of order in rapid sequences of tones J Exp Psychol 1971 89 2 244 249 5567132 10.1037/h0031163

[B2] Bregman AS Auditory streaming is cumulative J Exp Psychol Hum Percept Perform 1978 4 3 380 387 681887 10.1037//0096-1523.4.3.380

[B3] Van Noorden L Temporal cocherence in the perception of tone sequences Eindhoven 1975 Eindhoven University of Technology, the Niederlands

[B4] Zwislocki J Theory of temporal auditory summation J Acoust Soc Am 1960 32 1046 1059 10.1121/1.1908276

[B5] Pressnitzer D Hupe JM Temporal dynamics of auditory and visual bistability reveal common principles of perceptual organization Curr Biol 2006 16 13 1351 1357 10.1016/j.cub.2006.05.054 16824924

[B6] Carlyon RP Cusack R Foxton JM Robertson IH Effects of attention and unilateral neglect on auditory stream segregation J Exp Psychol Hum Percept Perform 2001 27 1 115 127 11248927 10.1037//0096-1523.27.1.115

[B7] Hartmann WM JD Sream segregation and peripheral channeling Music Perception 1991 9 155 184

[B8] Carlyon RP How the brain separates sounds Trends Cogn Sci 2004 8 10 465 471 10.1016/j.tics.2004.08.008 15450511

[B9] Carlyon RP Micheyl C Deeks JM Moore BC Auditory processing of real and illusory changes in frequency modulation (FM) phase J Acoust Soc Am 2004 116 6 3629 3639 10.1121/1.1811474 15658713

[B10] Carlyon RP Plack CJ Fantini DA Cusack R Cross-modal and non-sensory influences on auditory streaming Perception 2003 32 11 1393 1402 10.1068/p5035 14959799

[B11] Gockel H Moore BC Patterson RD Asymmetry of masking between complex tones and noise: the role of temporal structure and peripheral compression J Acoust Soc Am 2002 111 6 2759 2770 10.1121/1.1480422 12083211

[B12] Bee MA Klump GM Auditory stream segregation in the songbird forebrain: effects of time intervals on responses to interleaved tone sequences Brain Behav Evol 2005 66 3 197 214 10.1159/000087854 16127270

[B13] Brosch M Schreiner CE Time course of forward masking tuning curves in cat primary auditory cortex J Neurophysiol 1997 77 2 923 943 9065859 10.1152/jn.1997.77.2.923

[B14] Fishman YI Arezzo JC Steinschneider M Auditory stream segregation in monkey auditory cortex: effects of frequency separation, presentation rate, and tone duration J Acoust Soc Am 2004 116 3 1656 1670 10.1121/1.1778903 15478432

[B15] Fishman YI Reser DH Arezzo JC Steinschneider M Neural correlates of auditory stream segregation in primary auditory cortex of the awake monkey Hear Res 2001 151 1–2 167 187 11124464 10.1016/s0378-5955(00)00224-0

[B16] Gutschalk A Micheyl C Melcher JR Rupp A Scherg M Oxenham AJ Neuromagnetic correlates of streaming in human auditory cortex J Neurosci 2005 25 22 5382 5388 10.1523/JNEUROSCI.0347-05.2005 15930387 PMC1237040

[B17] Kanwal JS Medvedev AV Micheyl C Neurodynamics for auditory stream segregation: tracking sounds in the mustached bat's natural environment Network 2003 14 3 413 435 10.1088/0954-898X/14/3/303 12938765 PMC13330546

[B18] Micheyl C Tian B Carlyon RP Rauschecker JP Perceptual organization of tone sequences in the auditory cortex of awake macaques Neuron 2005 48 1 139 148 10.1016/j.neuron.2005.08.039 16202714

[B19] Micheyl C Carlyon RP Gutschalk A Melcher JR Oxenham AJ Rauschecker JP Tian B Courtenay Wilson E The role of auditory cortex in the formation of auditory streams Hear Res 2007 229 1–2 116 131 17307315 10.1016/j.heares.2007.01.007 PMC2040076

[B20] Snyder JS Alain C Sequential auditory scene analysis is preserved in normal aging adults Cereb Cortex 2007 17 3 501 512 16581981 10.1093/cercor/bhj175

[B21] Snyder JS Alain C Age-related changes in neural activity associated with concurrent vowel segregation Brain Res Cogn Brain Res 2005 24 3 492 499 10.1016/j.cogbrainres.2005.03.002 16099361

[B22] Snyder JS Alain C Picton TW Effects of attention on neuroelectric correlates of auditory stream segregation J Cogn Neurosci 2006 18 1 1 13 10.1162/089892906775250021 16417678

[B23] Snyder JS Carter OL Hannon EE Alain C Adaptation reveals multiple levels of representation in auditory stream segregation J Exp Psychol Hum Percept Perform 2009 35 4 1232 1244 19653761 10.1037/a0012741 PMC2726626

[B24] Wehr M Zador AM Synaptic mechanisms of forward suppression in rat auditory cortex Neuron 2005 47 3 437 445 10.1016/j.neuron.2005.06.009 16055066

[B25] Ulanovsky N Las L Nelken I Processing of low-probability sounds by cortical neurons Nat Neurosci 2003 6 4 391 398 10.1038/nn1032 12652303

[B26] Schadwinkel S Gutschalk A Activity associated with stream segregation in human auditory cortex is similar for spatial and pitch cues Cereb Cortex 2010 20 12 2863 2873 10.1093/cercor/bhq037 20237241

[B27] Schadwinkel S Gutschalk A Transient bold activity locked to perceptual reversals of auditory streaming in human auditory cortex and inferior colliculus J Neurophysiol 2011 105 5 1977 1983 10.1152/jn.00461.2010 21325685

[B28] Sussman E Steinschneider M Neurophysiological evidence for context-dependent encoding of sensory input in human auditory cortex Brain Res 2006 1075 1 165 174 10.1016/j.brainres.2005.12.074 16460703 PMC2846765

[B29] Kondo HM Kashino M Involvement of the thalamocortical loop in the spontaneous switching of percepts in auditory streaming J Neurosci 2009 29 40 12695 12701 10.1523/JNEUROSCI.1549-09.2009 19812344 PMC6665088

[B30] Beauvois MW Meddis R Computer simulation of auditory stream segregation in alternating-tone sequences J Acoust Soc Am 1996 99 4 Pt 1 2270 2280 8730073 10.1121/1.415414

[B31] Beauvois MW Meddis R Time decay of auditory stream biasing Percept Psychophys 1997 59 1 81 86 10.3758/BF03206850 9038410

[B32] Pressnitzer D Sayles M Micheyl C Winter IM Perceptual organization of sound begins in the auditory periphery Curr Biol 2008 18 15 1124 1128 10.1016/j.cub.2008.06.053 18656355 PMC2559912

[B33] Elhilali M Ma L Micheyl C Oxenham AJ Shamma SA Temporal coherence in the perceptual organization and cortical representation of auditory scenes Neuron 2009 61 2 317 329 10.1016/j.neuron.2008.12.005 19186172 PMC2673083

[B34] Snyder JS Carter OL Lee SK Hannon EE Alain C Effects of context on auditory stream segregation J Exp Psychol Hum Percept Perform 2008 34 4 1007 1016 18665741 10.1037/0096-1523.34.4.1007

[B35] Snyder JS Holder WT Weintraub DM Carter OL Alain C Effects of prior stimulus and prior perception on neural correlates of auditory stream segregation Psychophysiology 2009 46 6 1208 1215 10.1111/j.1469-8986.2009.00870.x 19674396

[B36] Hillyard SA Hink RF Schwent VL Picton TW Electrical signs of selective attention in the human brain Science 1973 182 108 177 180 10.1126/science.182.4108.177 4730062

[B37] Hillyard SA Electrical and magnetic brain recordings: contributions to cognitive neuroscience Curr Opin Neurobiol 1993 3 2 217 224 10.1016/0959-4388(93)90213-I 8513235

[B38] Cusack R Deeks J Aikman G Carlyon RP Effects of location, frequency region, and time course of selective attention on auditory scene analysis J Exp Psychol Hum Percept Perform 2004 30 4 643 656 15301615 10.1037/0096-1523.30.4.643

[B39] Snyder JS Alain C Toward a neurophysiological theory of auditory stream segregation Psychol Bull 2007 133 5 780 799 17723030 10.1037/0033-2909.133.5.780

[B40] Bregman AS Ahad PA Crum PA O'Reilly J Effects of time intervals and tone durations on auditory stream segregation Percept Psychophys 2000 62 3 626 636 10.3758/BF03212114 10909253

[B41] Rogers WL Bregman AS An experimental evaluation of three theories of auditory stream segregation Percept Psychophys 1993 53 2 179 189 10.3758/BF03211728 8433916

[B42] Cowan N On short and long auditory stores Psychol Bull 1984 96 341 370 6385047

[B43] Grossheinrich N Kademann S Bruder J Bartling J Von Suchodoletz W Auditory sensory memory and language abilities in former late talkers: a mismatch negativity study Psychophysiology 2010 47 5 822 830 20409011 10.1111/j.1469-8986.2010.00996.x

[B44] Copeland D Radvansky G Working memory and syllogistic reasoning Q J Exp Psychol A 2004 57 8 1437 1457 15513254 10.1080/02724980343000846

[B45] Tesche CD Uusitalo MA Ilmoniemi RJ Huotilainen M Kajola M Salonen O Signal-space projections of MEG data characterize both distributed and well-localized neuronal sources Electroencephalogr Clin Neurophysiol 1995 95 3 189 200 10.1016/0013-4694(95)00064-6 7555909

[B46] Loftus GR Masson MEJ Using confidence intervals in within-subject designs Psychon Bull Rev 1994 47 1 476 490 10.3758/BF03210951 24203555

[B47] Chait M Simon JZ Poeppel D Auditory M50 and M100 responses to broadband noise: functional implications Neuroreport 2004 15 16 2455 2458 10.1097/00001756-200411150-00004 15538173

[B48] McEvoy L Makela JP Hamalainen M Hari R Effect of interaural time differences on middle-latency and late auditory evoked magnetic fields Hear Res 1994 78 2 249 257 10.1016/0378-5955(94)90031-0 7982817

[B49] Makela JP Hamalainen M Hari R McEvoy L Whole-head mapping of middle-latency auditory evoked magnetic fields Electroencephalogr Clin Neurophysiol 1994 92 5 414 421 10.1016/0168-5597(94)90018-3 7523085

[B50] Levanen S Sams M Disrupting human auditory change detection: Chopin is superior to white noise Psychophysiology 1997 34 3 258 265 10.1111/j.1469-8986.1997.tb02396.x 9175440

[B51] Pantev C Okamoto H Ross B Stoll W Ciurlia-Guy E Kakigi R Kubo T Lateral inhibition and habituation of the human auditory cortex Eur J Neurosci 2004 19 8 2337 2344 10.1111/j.0953-816X.2004.03296.x 15090060

[B52] Coenen A Modelling of auditory evoked potentials of human sleep-wake states Int J Psychophysiol 2011 In press 10.1016/j.ijpsycho.2011.10.010 22133997

[B53] Tian S Qi H Wang J Cai J Ma Y Differential amplitude modulation of auditory evoked cortical potentials associated with brain state in the freely moving rhesus monkey Neurosci Lett 2002 331 3 159 162 10.1016/S0304-3940(02)00886-8 12383921

[B54] Roberts TP Poeppel D Latency of auditory evoked M100 as a function of tone frequency Neuroreport 1996 7 6 1138 1140 10.1097/00001756-199604260-00007 8817518

[B55] Howard MF Poeppel D Hemispheric asymmetry in mid and long latency neuromagnetic responses to single clicks Hear Res 2009 257 1–2 41 52 19647788 10.1016/j.heares.2009.07.010 PMC2766856

[B56] Calford MB Semple MN Monaural inhibition in cat auditory cortex J Neurophysiol 1995 73 5 1876 1891 7623087 10.1152/jn.1995.73.5.1876

[B57] Draganova R Ross B Borgmann C Pantev C Auditory cortical response patterns to multiple rhythms of AM sound Ear Hear 2002 23 3 254 265 10.1097/00003446-200206000-00009 12072617

